# Health Risk Assessment of Exposure to Air Pollutants Exceeding the New WHO Air Quality Guidelines (AQGs) in São Paulo, Brazil

**DOI:** 10.3390/ijerph20095707

**Published:** 2023-05-02

**Authors:** Caroline Fernanda Hei Wikuats, Thiago Nogueira, Rafaela Squizzato, Edmilson Dias de Freitas, Maria de Fatima Andrade

**Affiliations:** 1 Departamento de Ciências Atmosféricas, Instituto de Astronomia, Geofísica de Ciências Atmosféricas, Universidade de São Paulo, São Paulo 05508-090, Brazil; 2 Departamento de Saúde Ambiental, Faculdade de Saúde Pública, Universidade de São Paulo, São Paulo 01246-904, Brazil

**Keywords:** air pollutants, ambient air quality standards, health outcomes, AirQ+ software

## Abstract

We applied the AirQ+ model to analyze the 2021 data within our study period (15 December 2020 to 17 June 2022) to quantitatively estimate the number of specific health outcomes from long- and short-term exposure to atmospheric pollutants that could be avoided by adopting the new World Health Organization Air Quality Guidelines (WHO AQGs) in São Paulo, Southeastern Brazil. Based on temporal variations, PM_2.5_, PM_10_, NO_2_, and O_3_ exceeded the 2021 WHO AQGs on up to 54.4% of the days during sampling, mainly in wintertime (June to September 2021). Reducing PM_2.5_ values in São Paulo, as recommended by the WHO, could prevent 113 and 24 deaths from lung cancer (LC) and chronic obstructive pulmonary disease (COPD) annually, respectively. Moreover, it could avoid 258 and 163 hospitalizations caused by respiratory (RD) and cardiovascular diseases (CVD) due to PM_2.5_ exposure. The results for excess deaths by RD and CVD due to O_3_ were 443 and 228, respectively, and 90 RD hospitalizations due to NO_2_. Therefore, AirQ+ is a useful tool that enables further elaboration and implementation of air pollution control strategies to reduce and prevent hospital admissions, mortality, and economic costs due to exposure to PM_2.5_, O_3_, and NO_2_ in São Paulo.

## 1. Introduction

Air pollution is one of the greatest environmental risks to human health increasing morbidity and mortality and reducing life expectancy [1,2]. In 2019, air pollution ranked fourth as the world’s leading risk factor for early death causing 6.7 million deaths worldwide, of which 4.1 million were due to ambient air pollution [3,4,5,6].

According to the World Health Organization (WHO), in 2019, 37% of premature deaths related to outdoor air pollution occurred due to ischemic heart disease (IHD) and stroke, while 23 and 18% were caused by acute lower respiratory infections and chronic obstructive pulmonary disease (COPD), respectively, and 11% due to lung cancer (LC) [2]. Furthermore, global deaths (in millions of people) resulting from exposure to ambient air pollution have increased by 51% since 1990 and are estimated to double by 2050 if more relevant interventions do not occur [5,7].

Air pollution consists of both gaseous and particulate primary pollutants released directly into the atmosphere, such as nitrogen oxides (NO_x_), carbon monoxide (CO), and sulfur dioxide (SO_2_), as well as secondary pollutants formed in the atmosphere, including fine particulate matter (PM_2.5_) and ozone (O_3_). Particulate matter is the most studied pollutant presenting consistent evidence of adverse health effects [8,9]. Exposure to both coarse and fine particles (PM_10_ and PM_2.5_, respectively) is harmful to health, but the latter represents the most robust predictor of mortality from lung cancer, respiratory, cardiovascular, and other diseases [1,3]. Numerous studies also report a relationship between exposure to gaseous pollutants, such as O_3_, and increased morbidity and mortality [5].

In 2019, more than 90% of the world’s population was living in areas, mainly in low- and middle-income countries (e.g., Brazil), where ambient air pollution concentrations surpassed the 2005 WHO guideline for PM_2.5_ (annual mean: 10 µg m^−3^) [3]. The WHO Air Quality Guidelines (AQGs) provide evidence-based guidance for air pollutant levels to reduce exposure and protect public health. In 2021, the AQGs were updated due to the ongoing risk of air pollution to human health, with more restrictive concentrations for pollutants such as PM_2.5_ (annual mean: from 10 to 5 µg m^−3^), PM_10_, CO, and nitrogen dioxide (NO_2_) [10,11].

In Brazil, despite the reductions in primary pollutant concentrations achieved through the implementation of air pollution control programs in recent years, several levels remain above the WHO AQGs. Additionally, although public policies such as the Program for the Control of Air Pollution Emissions by Motor Vehicles (PROCONVE, established in 1986) have led to a decrease in vehicular emissions, the growth of the vehicle fleet (as reported by Nogueira et al. [12]) has offset these gains. The concentrations of secondary pollutants such as PM_2.5_ and O_3_ remain a concern and are not yet effectively controlled, as noted by Andrade et al. [13]. Further studies on outdoor exposure to mainly secondary pollutants are necessary to develop or adjust policies, regulate their concentrations, improve air quality, and potentially reduce adverse health effects.

Moreover, there is a scarcity of studies conducted in Brazil focusing on the quantitative assessment of air pollution effects on hospitalizations and mortality. Considering existing research based on data from the 1990s and 2000s, a positive association was observed between Disability-Adjusted Life Years (DALYs) and air pollution in São Paulo adding up to 28,212 years annually and more than 5000 deaths that could be prevented if PM_2.5_ levels were reduced to the 2005 WHO AQG of 10 µg m^−3^ [14,15]. These studies present relevant results related to air pollution and health effects in the Metropolitan Area of São Paulo (MASP), but it is also important to evaluate the outcomes with the new WHO AQGs, taking into account the reduction in the levels of air pollutants observed recently. To the best of our knowledge, this is the first study assessing the health risks of air pollutants in the region using AirQ+, which is a tool for quantifying the health burden and impact of air pollution developed by the WHO Regional Office for Europe [16].

The progress toward achieving the Sustainable Development Goals (SDG) outlined in the 2030 Agenda has been insufficient and unequal globally [17]. In Brazil, these inadequacies are apparent in the statistical monitoring of the 2030 Agenda, which fails to report data on mortality rates caused by household and outdoor air pollution. To address this issue, we employed the AirQ+ model to quantify the number of health outcomes that could be prevented by adopting the new WHO AQGs for long- and short-term exposure to PM_2.5_, NO_2_, and O_3_ in São Paulo, Brazil, based on their temporal variations.

## 2. Materials and Methods

### 2.1. Study Site

São Paulo is South America’s largest and most developed and industrialized region. It has an estimated population of approximately 12.4 million individuals, a territorial unit area of 1521 km^2^, a population density of around 7398 inhabitants.km^−2^, and a Human Development Index (HDI) of 0.805 [18]. In addition, it has a Gross Domestic Product (GDP) of around USD 150 billion, corresponding to 33% from the state of São Paulo and 58% from the MASP [19,20].

According to Köppen’s classification, the region presents a humid subtropical climate (Cwa) with dry and cool winters (temperatures below 18 °C) and wet and warm summers (temperatures above 22 °C) [21]. São Paulo is surrounded by hills of 1200 m and is located on a plateau at 860 m above sea level. The frequent occurrence of subsidence layers and thermal inversion makes the dispersion of pollutants difficult, especially during winter [22].

**Figure 1:**
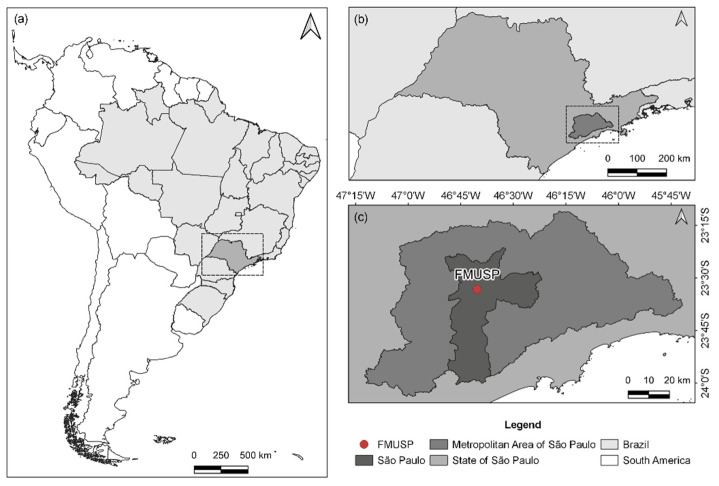
Location of Brazil and the state of São Paulo (dashed line rectangle) (**a**), the Metropolitan Area of São Paulo (MASP) (dashed line rectangle) within the state (**b**), the city of São Paulo and the School of Medicine of the University of São Paulo (FMUSP) (**c**).

Furthermore, São Paulo has a fleet of around 8.9 million vehicles responsible for the emissions of 96% of CO, 73% of hydrocarbons (HC), 65% of NO_x_, 40% of PM, and 11% of SO_x_ [23,24]. We carried out measurements with a mobile laboratory at the School of Medicine of the University of São Paulo (FMUSP) (23°33′16.2″ S, 46°40′19.7″ W) (Figure 1), where the influence of emissions from mobile sources, such as light- and heavy-duty vehicles (LDVs and HDVs, respectively), is significant.

### 2.2. Data Sampling and Analysis

We performed the sampling campaign of air pollutants with the Mobile Laboratory for Research and Monitoring of Air Quality (LuMIAR) at the FMUSP continuously between 15 December 2020 and 17 June 2022 (a total of 550 days). LuMIAR is equipped with monitors for measuring PM_2.5_, PM_10_, SO_2_, CO, NO_2_, and O_3_, and the data are reported on a 1-h average. In Table 1, we present information from the instruments considered for this study [25]. Concentration units for PM_2.5_, PM_10_, SO_2_, NO_2,_ and O_3_ are presented in µg m^−3^, while CO is presented in mg m^−3^ to compare with the AQGs. The Weather Station of the Institute of Astronomy, Geophysics, and Atmospheric Sciences of the University of São Paulo provided the meteorological variables (air temperature, precipitation, wind speed, and wind direction).

We performed all the data organization and statistical analyses in the R Software (version 4.0.3) and used the openair package (version 2.10-0) to plot time series, temporal variations, and wind rose graphs for the pollutants and meteorological variables [26,27]. We used the AirQ+ software tool (version 2.1.1) to conduct health risk assessments for long- and short-term effects [16] based on temporal trends for PM_2.5_, NO_2_, and O_3_. It can be applied for any region to evaluate how much a specific health outcome is attributable to certain air pollutant concentrations and the health effects if levels change in the future compared to the present. AirQ+ calculations were based on methodologies and concentration-response functions determined by epidemiological studies available up to 2013 and their meta-analysis [28]. Some limitations of the software include the following: calculations do not consider multiple exposure cases or multi-pollutant scenarios; it uses ambient data as a proxy indicator of population exposure; and “morbidity estimates present low reliability due to difficult conformity in the assessment of health outcomes related to hospital admissions” [28].

We obtained population data for the city of São Paulo from the Brazilian Institute of Geography and Statistics (IBGE) [18], and mortality and hospital admission data from the Department of Informatics of the Brazilian Unified Health System (DATASUS) [29]. We considered the period from 1 January to 31 December 2021, for the incidence per 100,000 inhabitants at risk per year based on the mortality and hospitalization statistics, as it was the only complete year available in the dataset. Mortality data included LC, COPD, IHD, and stroke information for different age groups. On the other hand, hospitalization and mortality data considered respiratory (RD) and cardiovascular diseases (CVD) for the total population.

Furthermore, we used 24-h mean values for the entire year, location area size, total population, the population at risk, incidence per 100,000 inhabitants, and cut-off concentration as the input data in the software. For long-term exposure, we applied the annual WHO AQGs that denoted “the lower end of the range in which significant effects on survival have been observed” [30]. On the other hand, the 24-h mean WHO AQGs were considered for short-term effects. The AirQ+ software already included default relative risk (RR) values. We utilized the log-linear and the Global Burden of Disease (GBD) 2015/2016 (integrated function 2016 vs. 2005 WHO AQG value) methods to estimate the results for the pollutants. The optional input data included latitude, longitude, and location identification. We assessed different health-related results using this software tool, including the attributable proportion of cases, the number of attributable cases, the number of attributable cases per 100,000 population at risk, the proportion of cases in the pollutant concentration range, and the cumulative distribution by air pollutant concentration [16,31,32]. We also used Origin (version 2020) to plot bar charts for the attributable cases of mortality and hospital admissions.

## 3. Results and Discussion

### 3.1. Pollutant Concentrations and Meteorological Variables Overview

**Figure 2:**
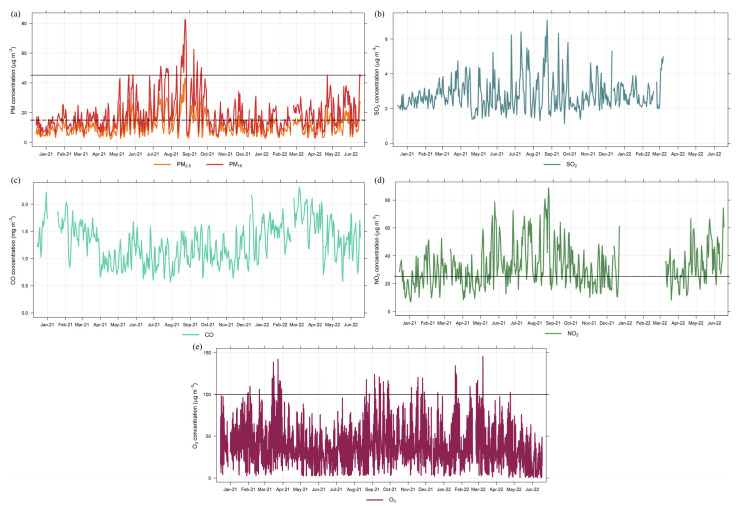
Twenty-four-hour time series for PM_2.5_ and PM_10_ (**a**), SO_2_ (**b**), CO (**c**), and NO_2_ (**d**), and 8-h time series for O_3_ (**e**) during the sampling period. The black horizontal lines indicate the WHO Air Quality Guidelines. 24-h mean for PM_2.5_: 15 µg m^−3^, PM_10_: 45 µg m^−3^, NO_2_: 25 µg m^−3^; and 8-h mean for O_3_: 100 µg m^−3^.

Descriptive statistics and time series for the pollutants measured at the FMUSP are shown in Table 2 and Figure 2, respectively. Mean values for all pollutants were similar in 2021, 2022, and throughout the entire sampling period (15 December 2020 to 17 June 2022) (Table 2). Abe and Miraglia [15] reported concentrations of 21 ± 10 µg m^−3^ for PM_2.5_, 36 ± 17 µg m^−3^ for PM_10_, and 83 ± 36 µg m^−3^ for O_3_ between 2009 and 2011 in São Paulo. Compared with the overall mean values obtained at the FMUSP, these three pollutants observed significant decreases of 47.1, 47.8, and 55.9% over ten years.

Nevertheless, PM_2.5_, PM_10_, NO_2_, and O_3_ still exceeded the 2021 WHO AQGs on 117 (21.3%), 26 (4.7%), 299 (54.4%), and 171 (31.1%) days, mainly during wintertime (June to September 2021), considering the 550 days of the sampling campaign (Figure 2). For NO_2_, 54.4% of the data exceeded the AQG for the 24-h mean, while it was not surpassed when considering the 1-h mean (not shown). The graphs for SO_2_ and CO do not include black horizontal lines indicating the WHO AQGs, as the values registered at the FMUSP were much lower. Over the years, an expressive reduction in SO_2_ concentrations in São Paulo has occurred due to the diminished sulfur content in vehicular and industrial fuels [33].

The WHO AQGs are much more restrictive than the Brazilian National Air Quality Standards (NAQS) recommended by the Brazilian National Environmental Council (CONAMA) Resolution 491/2018 [34] (Table 3), which was established considering the 2005 WHO AQGs as a reference. Nonetheless, the implementation criteria consider intermediate NAQS, i.e., standards determined as temporary values to be completed in phases, although the period of use of each one before reaching the final AQGs (from 2005) has not been specified. Therefore, the values for intermediate phase 1 are currently in effect. Comparing our results with the NAQS, none of the pollutants surpassed the limits (not shown), which highlights the differences between the Brazilian legislation and the WHO recommendations. Hence, there is a need for monitoring the concentrations of atmospheric pollutants in the region and for more effective policies to implement restrictive limits following the WHO guidelines, reducing the delay that is currently observed.

Historically, cities in Brazil (including São Paulo) have suffered from rampant violations of AQGs, particularly in the 1970s and 1980s, due to a lack of control and regulation of air pollution sources. However, following international initiatives, governmental organizations have implemented various measures to address this issue, focusing on the reduction in emissions from mobile sources in the transport sector [13]. The most successful initiative was implementing the PROCONVE program, based on CONAMA Resolutions 18/1986 and 297/2002, effectively reducing concentrations of primary pollutants, such as CO, PM_10_, and SO_2_ [35,36]. The program was responsible for decreasing 90% of emissions from LDVs and 80% from HDVs based on enforceable legislation to promote national technological development in automobile engineering and methods and equipment for testing and measuring pollutant emissions, to encourage the large-scale use of biofuels, and to reduce the sulfur content in fuels [13,33,35,36]. For example, it resulted in the introduction of flex-fuel LDVs (powered by gasohol, ethanol, or any mixture of both) considering new exhaust systems and catalytic converters, and the addition of biodiesel to diesel for HDVs. Since 2012, post-treatment with selective catalytic reduction (SCR) systems for the NO_x_ emissions of HDVs has also been mandatory [12].

In 1990, CONAMA Resolution 003/1990 established NAQS considering the need to monitor and control air pollutants in Brazil, which was then repealed by CONAMA Resolution 491/2018 to follow the 2005 WHO AQGs as mentioned above. Furthermore, the city of São Paulo implemented driving restrictions in the 1990s based on the last digits of the license plate of cars on pre-established days during peak hours (from 7 to 10 h and from 17 to 20 h). This initiative was introduced to decrease air pollution emissions and alleviate traffic congestion [13,33].

Regarding industrial emissions in the MASP, currently, there is a small contribution to the concentrations of air pollutants due to a moving process of point sources to other cities, which had fewer restrictions to install potentially polluting industries, that occurred in the 1980s and 1990s. On the other hand, policies have been implemented to reduce industrial emissions since the 1980s in the state of São Paulo. For example, the change from oil-fired to electric boilers to generate energy for industries was remarkable in reducing SO_2_ ambient concentrations. In 2007, another important action was to institute rules for new facilities considering the local level of regular pollutants (CO, NO_2_, O_3_, PM_10_, total particulate matter, SO_2_, and smoke) [13,33].

Nogueira et al. [12] reported reductions of 4.9 and 5.1% per year for CO and of 5.5 and 4.2% per year for NO_x_ from 2001 to 2018 in São Paulo considering emission factors for LDVs and HDVs, respectively. The authors discussed that, although overdue, the vehicular emission regulations adopted in Brazil have significantly improved air quality in the MASP. From 1996 to 2009, a downward trend was also observed for all pollutants (CO, NO_x_, SO_2_, and PM_10_) monitored by the Environmental Company of the State of São Paulo (CETESB), except for O_3_ [33]. Therefore, despite the reductions in primary pollutants levels achieved with air pollution regulation programs, controlling the concentrations and emissions of secondary pollutants such as PM_2.5_ and O_3_ is the greatest challenge currently faced by governmental organizations in São Paulo and Brazil.

**Figure 3:**
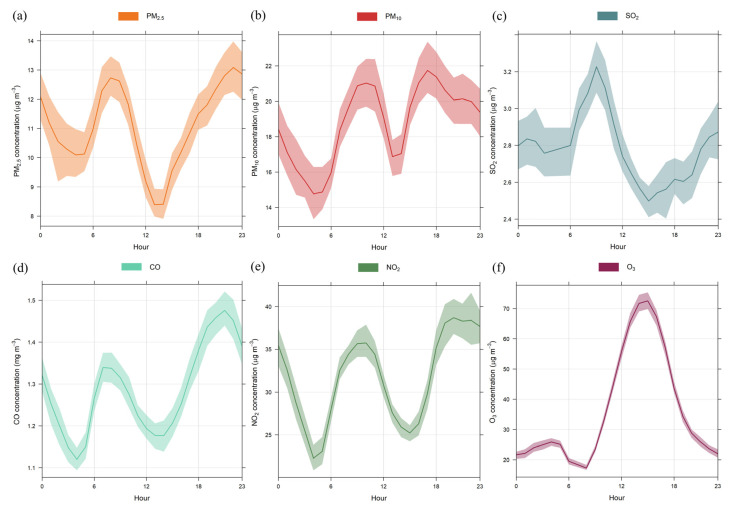
Hourly variations for PM_2.5_ (**a**), PM_10_ (**b**), SO_2_ (**c**), CO (**d**), NO_2_ (**e**), and O_3_ (**f**) during the sampling period. The shaded areas represent the 95% confidence intervals of the mean.

The hourly time variations of pollutants are shown in Figure 3 for the entire sampling period (hours are shown as local time). Except for O_3_, the pollutants presented similar hourly profiles, with two peaks (from 8 to 11 h and 19 to 23 h) due to emissions from mobile sources with increased traffic on the roads close to the sampling site. The peak associated with the nighttime rush hour indicated larger time variability among the pollutants, mainly for PM_10_ and CO, which presented peaks earlier (from 16 to 18 h) and later (between 23 and 1 h) in the day, respectively. In addition, the Planetary Boundary Layer (PBL) stability in the evening hampers pollutants’ dispersion, maintaining high concentrations during this period. Moreover, the hourly profiles follow the characteristics already observed in São Paulo [22,33].

For O_3_, the highest concentrations occurred between 14 and 16 h, a different behavior from the other pollutants due to photochemical reactions between O_3_ precursors (NO_x_ and volatile organic compounds—VOCs) and solar radiation. The chemistry of NO_x_ at night differs from that observed during the day, as NO_2_ photolysis does not occur, i.e., NO present in the atmosphere at night can rapidly react with O_3,_ and nearly all NO_x_ is converted into NO_2_ [37]. As also observed by Carvalho et al. and Massambani and Andrade [33,38], between 3 and 4 h (Figure 3), a secondary peak appeared due to horizontal and vertical transport from other regions as there is no O_3_ formation during the night [33,39].

In Figure S1 of the Supplementary Material, July, August, and September presented the highest monthly averages for PM_2.5_, PM_10_, SO_2_, and NO_2_, corresponding to winter in Brazil, a characteristic period of higher levels of air pollution due to stable atmospheric conditions and lower precipitation rates. It is possible to observe great variability in the CO data (Figure S1), with the highest concentrations in January and February at the FMUSP, which was not expected but probably occurred due to a specific local source still under investigation.

**Figure 4:**
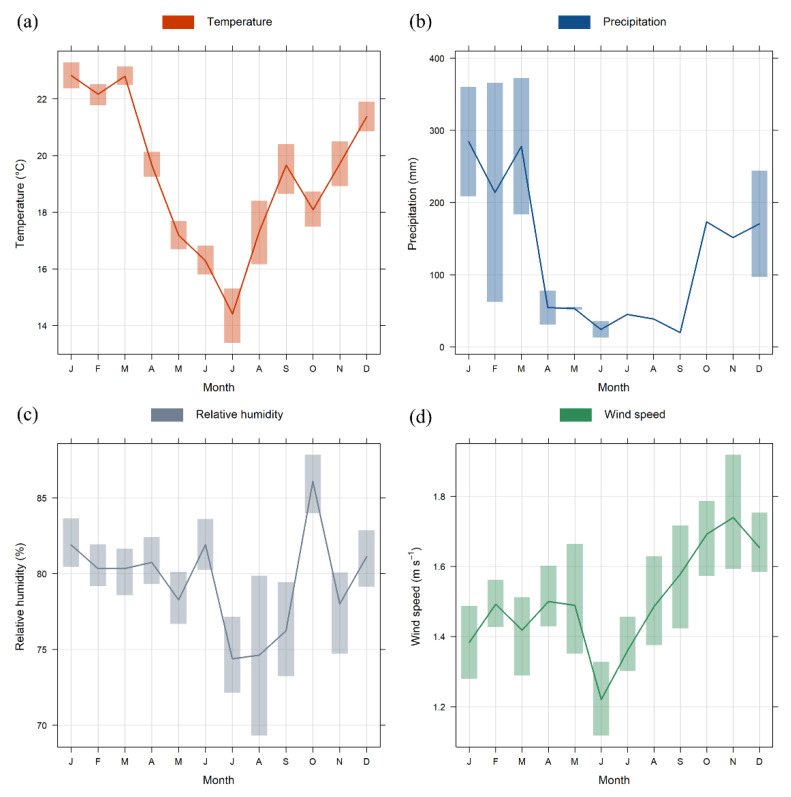
Monthly variations for temperature (**a**), precipitation (**b**), relative humidity (**c**), and wind speed (**d**) during the sampling period. The shaded areas represent the 95% confidence intervals of the mean.

These results agree with the local meteorological conditions (Figure 4 and Figure S2). The mean temperature values over the entire sampling period were 19.7 ± 3.4 °C, while precipitation was recorded on 266 days with a maximum of 71.2 mm/day. As expected, April to September (fall and winter) presented the lowest precipitation rates, since São Paulo has dry and cool winters [18]. The relative humidity was 79.9 ± 8.1%, and the wind speed was 1.5 ± 0.5 m s^−1^ with a maximum of 4.9 m s^−1^. Wind direction showed a consistent pattern coming from the southeast and northeast more than 50 and 30% of the time. These meteorological data follow the climate normal (1991–2020) for São Paulo according to information from the Brazilian National Institute of Meteorology (INMET) (not shown).

Our analysis revealed a decrease in the concentrations of primary pollutants during the weekend, mainly on Sunday (Figure S1), as the number of vehicles in circulation can decrease up to 70% compared to weekdays, according to data from the Traffic Engineering Company of São Paulo [40]. On the other hand, O_3_ levels were relatively higher during the weekends, a trend already documented in the region and associated with a VOC-limited atmosphere in urban areas [41,42]. This is because the substantial reduction in NO_x_ emissions from HDVs on weekends leads to an increase in O_3_ levels [25,37].

### 3.2. Health Risk Assessment

The main impact assessment results from AirQ+ are relative risk (RR) (and 95% confidence intervals—CI), attributable cases per 100,000 population at risk (B + c), and attributable cases (N + c).

#### 3.2.1. Outcomes from PM_2.5_ Exposure

**Figure 5:**
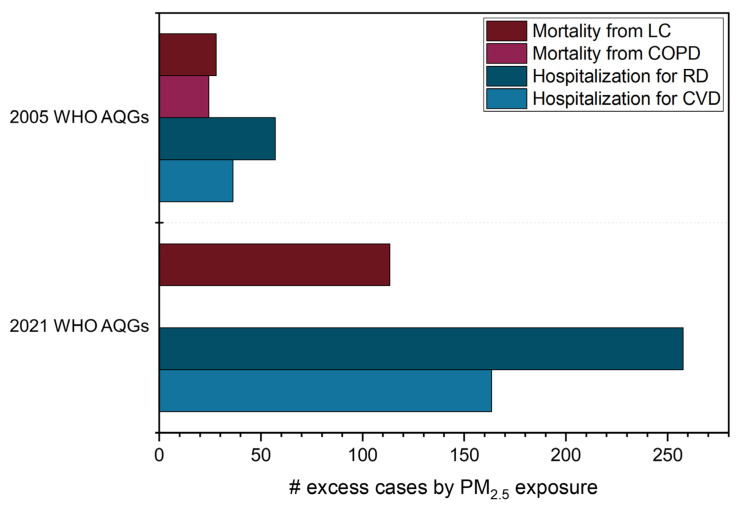
Attributable cases of mortality and hospital admissions caused by PM_2.5_ exposure considering the 2005 and 2021 WHO Air Quality Guidelines. LC—lung cancer; COPD—chronic obstructive pulmonary disease; RD—respiratory disease; CVD—cardiovascular disease.

Regarding long-term health effects, we evaluated the following outcomes: mortality from LC and COPD. The RR of mortality from LC was 1.4 (95% CI: 0.6–2.1%) and 5.8% (95% CI: 2.6–9.0%) higher for people exposed to the annual 11.6 µg m^−3^ registered in São Paulo compared to the annual WHO AQGs of 10 and 5 µg m^−3^, respectively (Table 4). In addition, mortality due to LC attributable to long-term exposure to PM_2.5_ concentrations above the 2005 WHO AQG scenario was 28 cases. For the 2021 WHO AQG, it was 113 cases (Figure 5), representing a 300% difference. Therefore, reducing PM_2.5_ values in São Paulo from 11.6 to 5 µg m^−3^, as recommended by the WHO, could prevent 113 deaths from lung cancer in the region annually. The RR of mortality from COPD in São Paulo was 1.7% (95% CI: 0.9–2.6%) higher for people exposed to the annual 11.6 µg m^−3^ than 10 µg m^−3^. Additionally, 24 excess mortality cases for this health outcome were observed considering long-term exposure to PM_2.5_ above the 2005 annual WHO AQG (Figure 5).

We estimated the following outcomes concerning short-term health effects: hospital admissions for RD and CVD. For the former, excess hospitalizations increased from 57 to 258 when comparing the 2005 and 2021 WHO AQGs results (Figure 5 and Table 4). For the latter, the values were 36 and 163 excess hospital admission cases caused by PM_2.5_ concentrations over the 2005 and 2021 24-h mean WHO AQGs, respectively. This indicates an increase of 350% in both health outcomes for the number of excess hospitalizations when comparing the WHO AQGs (from 2005 and 2021). On the other hand, the results were diluted in age groups for IHD and stroke; hence, the excess mortality varied from 0.36 (35–39 years old) to 3.65 cases (60–64 years old) and from 0.05 (25–29 years old) to 3.86 cases (80–84 years old), respectively, for long-term effects (Table 5).

#### 3.2.2. Outcomes from O_3_ and NO_2_ Exposure

For O_3_, we calculated the sum of ozone means over 35 ppb (SOMO35) (or 70 µg m^−3^), and added it as the input concentration, as indicated by the WHO [16]. A decrease in peak O_3_ values in several regions in Europe occurred during the 1990s, but no trend was observed in the sum of maximum 8-h O_3_ levels over 35 ppb (70 µg m^−3^). Therefore, SOMO35 is a metric for health impact assessment with a recommended cut-off value of 70 µg m^−3^ due to a statistically significant increase observed in mortality risk estimates considering O_3_ concentrations above 50–70 µg m^−3^. In addition, more consistent atmospheric model estimates were available for results above 70 µg m^−3^ [43].

**Figure 6:**
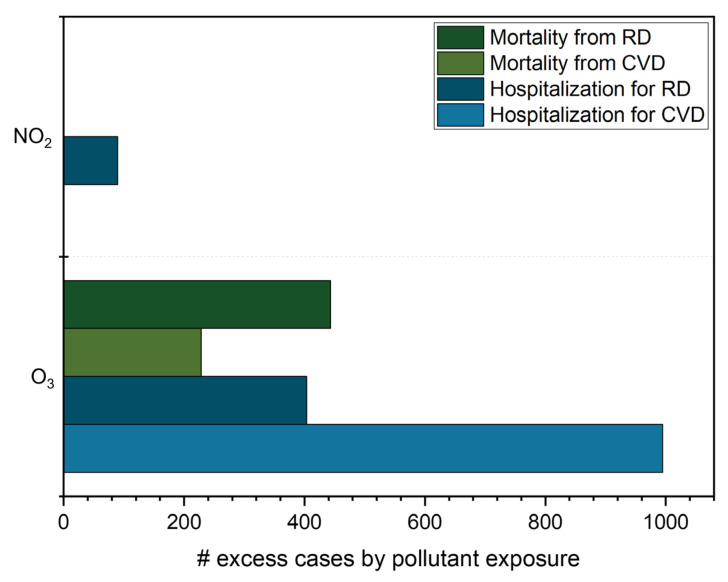
Attributable cases of mortality and hospital admissions caused by O_3_ and NO_2_ exposure considering the sum of ozone means over 35 ppb (SOMO35) and the 2021 WHO Air Quality Guideline, respectively. RD—respiratory disease; CVD—cardiovascular disease.

RD caused 443 and 93 excess annual mortality cases for long- and short-term effects, respectively, while for hospital admissions, the result was 404 incidents. O_3_ was also attributable to 228 and 995 excess mortality and hospitalization results for CVD, respectively (Figure 6 and Table 6).

For NO_2_, there were 90 excess hospital admission incidents due to RD (Figure 6 and Table 7). Thus, compliance with the 2021 WHO AQG for NO_2_ could prevent 90 hospitalizations derived from respiratory diseases annually in São Paulo. Notably, this number is probably underestimated since there may be a high rate of non-hospitalization care due to respiratory symptoms. It was not possible to compare with values from 2005, as this metric was introduced in 2021. All these results indicate that better public policymaking for air quality in São Paulo and other large cities in Brazil is necessary to reduce the number of deaths and hospital admissions due to exposure to air pollutants.

#### 3.2.3. Discussion of Health Outcomes Results

The well-known adverse health effects caused by PM_2.5_ may be aggravated by exposure to O_3_, which may cause lung epithelial damage and inflammatory response resulting in susceptibility to various infections [15,44]. Other epidemiologic studies also have shown significant health outcomes due to exposure to O_3_, such as toxic effects on pulmonary tissues and mortality from respiratory and cardiovascular diseases [15,45]. For NO_2_, several studies suggest that it may increase the risk for all-cause, respiratory, and cardiovascular mortality, besides asthma [46,47,48,49].

Other studies also evaluated the health effects caused by exposure to pollutants in different parts of the world using AirQ+ [31,50,51,52,53,54]. Kliengchuay et al. [50] reported 125, 27, and 26 deaths caused by COPD, IHD, and stroke due to long-term exposure to PM_2.5_ in Ratchaburi, Thailand, where the annual mean was 26.9 ± 18.7 µg m^−3^. The estimated number of premature deaths in Tehran, Iran, varied from 397 to 419 for IHD and from 86 to 102 for LC between 2016 and 2018, with mean PM_2.5_ concentrations from 29.4 ± 16.1 to 31.6 ± 16.2 µg m^−3^ [51]. Therefore, the results differ between studies depending on the concentrations registered in each region.

To the best of our knowledge, this is the first study assessing the health risks of air pollutants in São Paulo with AirQ+. However, several epidemiological studies have been conducted in the area to determine the adverse health effects of air pollution on the population based on acute effects in terms of hospital admissions, emergency room visits, and mortality in children and the elderly. According to Miraglia et al. [14], the key results of these studies showed a positive association even with concentrations below the AQGs. The life expectancy reduction due to air pollution was four years for RD, ten years for CVD, and 19 years for children with RD [14]. Health problems also generate high costs for the public health system. In the same study, Miraglia et al. [14] reported that the total health cost due to air pollution in São Paulo was more than USD 3.2 million. Nevertheless, these costs were not estimated in this investigation, which would be relevant to include in future analyses.

Abe and Miraglia [15] used the APHEKOM model and air pollutant levels between 2009 and 2011 to estimate that São Paulo could prevent more than 1500 cardiovascular and respiratory hospitalizations annually if a PM_10_ concentration of 20 µg m^−3^ (2005 WHO AQG) was reached. In addition, more than 5000 deaths could be postponed if PM_2.5_ levels were reduced to the 2005 WHO AQG of 10 µg m^−3^. The authors reported that life expectancy could increase by 15.8 months, corresponding to 266,486 life years gain and saving USD 15.1 billion annually. For O_3_, more than 50 respiratory hospital admissions could be avoided (population ≥ 15 years old) following the 2005 WHO AQG (average daily maximum 8-h mean: 100 µg m^−3^) [15]. In general, we presented lower results compared to the ones reported by Abe and Miraglia [15], although both studies were conducted in São Paulo; however, some significant aspects between the analyses caused this divergence. We used the WHO’s AirQ+ model (vs. APHEKOM by Abe and Miraglia [15]), which resulted in variations due to slightly different methodologies. Moreover, average concentrations between 2009 and 2011 [15] were higher than in 2021 (21 ± 10 vs. 11.6 ± 7.4 µg m^−3^ for PM_2.5_, 36 ± 17 vs. 19.5 ± 13.3 µg m^−3^ for PM_10_, and 83 ± 36 vs. 37.9 ± 21.8 µg m^−3^ for O_3_) as downward trends in atmospheric pollutants have been observed in the MASP due to vehicular emission regulations adopted in Brazil.

In this study, we only investigated PM_2.5_, O_3_, and NO_2_ due to the unavailability of robust data on the incidence of chronic bronchitis in adults, asthma symptoms in asthmatic children, and the prevalence of bronchitis in children required for the analysis of PM_10_ in the software. Additionally, AirQ+ does not have the option to evaluate CO and SO_2_. Hence, we used mortality and hospital admission data, which are less robust and more heterogeneous than the former. Mortality data are the best option for health risk assessments because they are accessible from high-quality records in São Paulo, reliable, and not subject to classification errors [15].

It is noteworthy that we obtained mortality and hospitalization data for São Paulo from DATASUS, which is a website for collecting, processing, and disseminating health information [29]. The Brazilian Ministry of Health classified COVID-19 as a severe acute respiratory syndrome (SARS), with another website for monitoring related cases and deaths that unfortunately presented remarkable numbers in the country [55]. In addition, the data for all health outcomes considered in our study did not show significant differences between 2017, 2018, 2019, 2020, and 2021 [29]. Thus, we believe there is a low probability of having COVID-19-related data among the information we used in the analyses.

Spatial averaging of the concentrations over an entire city may dampen the values and lower the mortality rates [51]. Therefore, using data from other monitoring stations in the city is important to have more robust results and identify trends related to the sources of air pollution emissions, which we suggest for future work. Nevertheless, monitoring stations do not correspond to the total exposure to air pollutants for all inhabitants in the region as it depends on different circumstances, such as indoor and outdoor activities and occupational exposure, among other factors [52].

This study also does not include other potential contributing factors to the relationship between air pollutants and hospitalization and mortality, such as body mass index, personal habits (e.g., smoking and drinking), physical activities, education, income, and medical history. Nonetheless, this study shows how many deaths and hospital admissions can be avoided in the area if more restrictive values for air pollutants are adopted, considering the 2021 WHO AQGs compared to those from 2005. It also should be noted that it accurately assesses health outcomes for different pollutants in the city of São Paulo, which exceed the 2021 WHO AQGs, and provides evidence to develop effective policies to enhance air quality and prevent health effects regarding hospitalizations and deaths.

## 4. Conclusions

In São Paulo, PM_2.5_, PM_10_, NO_2_, and O_3_ exceeded the new WHO AQGs, mainly during wintertime as July, August, and September presented the highest monthly averages for all pollutants, except for O_3_, which presents a different behavior due to photochemical reactions. Winter is a characteristic period of higher levels of air pollution in the region due to stable atmospheric conditions and lower precipitation rates. Wind speed presented low values, which also hindered the dispersion of pollutants.

The air pollutants considered in this study present consistent evidence of adverse health effects, i.e., mortality from lung cancer, stroke, and other respiratory and cardiovascular diseases. Considering pollutant concentrations and health data for the city of São Paulo, we estimated that reducing PM_2.5_ values could prevent 113 and 24 deaths from lung cancer and chronic obstructive pulmonary disease annually, respectively. In addition, it could avoid 258 and 163 hospitalizations caused by respiratory and cardiovascular diseases due to PM_2.5_ exposure. The results for excess respiratory and cardiovascular deaths due to O_3_ were 443 and 228, respectively, and 90 hospitalizations from respiratory diseases due to NO_2_. These data provide valuable information for local authorities to design and implement effective policies aimed at promoting a healthy environment.

In this study, we only considered temporal (and not spatial) trends for the concentrations of pollutants. Thus, future studies are needed to assess the differences in time variation, spatial distribution, and attributable proportion of hospitalizations and deaths in different regions of the city of São Paulo. Calculating the health costs due to air pollution would also add more important information for policymakers to analyze the cost-effectiveness of interventions.

AirQ+ is a useful tool that enables further elaboration and implementation of air pollution control strategies to reduce and prevent hospital admissions, mortality, and economic costs due to exposure to PM_2.5_, O_3_, and NO_2_ in São Paulo. The software is also helpful for national progress in implementing the 2030 Agenda and efficient public health regulations.

## Figures and Tables

**Table 1:** Information about the Mobile Laboratory for Research and Monitoring of Air Quality (LuMIAR) equipment.

Equipment.	Parameter	Model	Manufacturer	Unit
TEOM Continuous Ambient Particulate Monitor	PM_2.5_, PM_10_	1405	Thermo Fisher Scientific	µg m^−3^
SO_2_ Analyzer	SO_2_	43i	Thermo Fisher Scientific	µg m^−3^
Photoacoustic Gas Monitor	CO	Innova 1512	LumaSense Technologies	mg m^−3^
NO_x_ Analyzer	NO_2_	42i	Thermo Fisher Scientific	µg m^−3^
O_3_ Analyzer	O_3_	49i	Thermo Fisher Scientific	µg m^−3^

**Table 2:** Descriptive statistics of the pollutants sampled at the FMUSP between 15 December 2020 and 17 June 2022. Data uncertainty is expressed as the standard deviation (±values in parenthesis).

Pollutant	Overall Mean	2021 Mean	2022 Mean
PM_2.5_ (µg m^−3^)	11.1 (±6.8)	11.6 (±7.4)	10.5 (±5.3)
PM_10_ (µg m^−3^)	18.8 (±12.1)	19.5 (±13.3)	18.0 (±9.1)
SO_2_ (µg m^−3^)	2.8 (±1.0)	2.8 (±1.0)	2.9 (±1.0)
CO (mg m^−3^)	0.7 (±0.5)	1.2 (±0.4)	1.5 (± 0.4)
NO_2_ (µg m^−3^)	32.2 (±15.0)	32.2 (±15.4)	33.8 (±13.9)
O_3_ (µg m^−3^)	36.6 (±22.2)	37.9 (±21.8)	33.2 (±22.9)

**Table 3:** Air quality guidelines recommended by the WHO and CONAMA.

Pollutant		Averaging Time	WHO	CONAMA
PM_2.5_	(μg m^−3^)	Annual	5	20
		24-h	15	60
PM_10_		Annual	15	40
		24-h	45	120
SO_2_		24-h	40	125
CO	(mg m^−3^)		4	-
	(ppm)	8-h	9	9
NO_2_	(μg m^−3^)	Annual	10	60
		24-h	25	-
		1-h	200	260
O_3_		8-h	100	140

WHO—World Health Organization; CONAMA—Brazilian National Environmental Council (Conselho Nacional do Meio Ambiente).

**Table 4:** Impact assessment of health outcomes from long- and short-term exposure to PM_2.5_ considering the 2005 and 2021 WHO Air Quality Guidelines.

Health Endpoint	Timeframe	Age	2005 WHO AQG			2021 WHO AQG		
			RR	B + c	N + c	RR	B + c	N + c
Mortality from LC	Long-term	30+	1.0138 (1.0062–1.0210)	0.46 (0.21–0.70)	27.93 (12.76–42.32)	1.0584 (1.0262–1.0902)	1.89 (0.87–2.83)	113.41 (52.42–169.94)
Mortality from COPD		25+	1.0166 (1.0087–1.0258)	0.34 (0.18–0.53)	24.37 (12.93–37.53)	-	-	-
Hospital admissions for RD	Short-term	Total population	1.0008 (1.0000–1.0016)	0.51 (0.00–1.07)	57.07 (0.00–120.61)	1.0034 (1.0000–1.0073)	2.29 (0.00–4.84)	257.65 (0.00–544.74)
Hospital admissions for CVD			1.0004 (1.0001–1.0007)	0.32 (0.06–0.59)	36.20 (6.77–66.03)	1.0017 (1.0003–1.0030)	1.45 (0.27–2.65)	163.42 (30.54–298.01)

LC—lung cancer; COPD—chronic obstructive pulmonary disease; RD—respiratory disease; CVD—cardiovascular disease; RR—relative risk; B + c—estimated change of incidence (per 100,000) at a certain category of exposure; N + c—estimated number of cases attributable to a certain level of exposure.

**Table 5:** Impact assessment of health outcomes from long-term exposure to PM_2.5_ considering the 2005 WHO Air Quality Guideline.

Timeframe	Age	IHD			Stroke		
		RR	B + c	N + c	RR	B + c	N + c
Long-term	25–29	1.0220 (1.0122–1.0453)	0.04 (0.02–0.08)	0.43 (0.24–0.87)	1.0174 (1.0075–1.0274)	0.00 (0.00–0.01)	0.05 (0.02–0.08)
	30–34	1.0200 (1.0112–1.0418)	0.04 (0.02–0.08)	0.39 (0.22–0.80)	1.0162 (1.0076–1.0262)	0.02 (0.01–0.04)	0.24 (0.11–0.38)
	35–39	1.0185 (1.0100–1.0365)	0.04 (0.02–0.08)	0.36 (0.20–0.70)	1.0153 (1.0074–1.0249)	0.03 (0.01–0.04)	0.23 (0.11–0.37)
	40–44	1.0173 (1.0093–1.0377)	0.16 (0.09–0.35)	1.33 (0.72–2.83)	1.0140 (1.0064–1.0231)	0.09 (0.04–0.15)	0.75 (0.34–1.22)
	45–49	1.0159 (1.0088–1.0330)	0.16 (0.09–0.34)	1.22 (0.68–2.49)	1.0131 (1.0064–1.0211)	0.09 (0.05–0.15)	0.70 (0.34–1.12)
	50–54	1.0142 (1.0075–1.0298)	0.37 (0.20–0.76)	2.45 (1.31–5.07)	1.0119 (1.0055–1.0190)	0.25 (0.12–0.40)	1.68 (0.79–2.67)
	55–59	1.0128 (1.0073–1.0262)	0.40 (0.23–0.81)	2.21 (1.27–4.46)	1.0108 (1.0053–1.0178)	0.28 (0.14–0.46)	1.53 (0.76–2.51)
	60–64	1.0115 (1.0064–1.0233)	0.86 (0.48–1.73)	3.65 (2.03–7.32)	1.0097 (1.0048–1.0160)	0.62 (0.31–1.02)	2.61 (1.30–4.30)
	65–69	1.0102 (1.0058–1.0205)	1.07 (0.61–2.14)	3.25 (1.84–6.46)	1.0088 (1.0040–1.0145)	0.79 (0.36–1.29)	2.39 (1.08–3.90)
	70–74	1.0090 (1.0051–1.0191)	1.31 (0.74–2.75)	3.11 (1.76–6.53)	1.0078 (1.0038–1.0127)	1.12 (0.55–1.83)	2.67 (1.30–4.35)
	75–79	1.0077 (1.0048–1.0158)	1.56 (0.97–3.18)	2.67 (1.65–5.44)	1.0066 (1.0034–1.0111)	1.33 (0.69–2.23)	2.28 (1.18–3.81)
	80–84	1.0065 (1.0038–1.0118)	2.77 (1.65–5.01)	3.31 (1.97–5.99)	1.0057 (1.0027–1.0098)	3.23 (1.53–5.53)	3.86 (1.83–6.60)
	85–89	1.0054 (1.0031–1.0103)	4.83 (2.80–9.21)	2.76 (1.60–5.27)	1.0047 (1.0023–1.0078)	5.58 (2.70–9.24)	3.19 (1.54–5.29)
	90–94	1.0043 (1.0026–1.0081)	10.30 (6.33–19.52)	2.19 (1.35–4.14)	1.0038 (1.0018–1.0062)	12.17 (5.81–19.58)	2.58 (1.23–4.16)
	95+	1.0031 (1.0019–1.0056)	24.65 (14.88–44.00)	1.61 (0.97–2.87)	1.0028 (1.0013–1.0046)	29.05 (13.59–47.15)	1.90 (0.89–3.08)

IHD—ischemic heart disease; RR—relative risk; B + c—estimated change of incidence (per 100,000) at a certain category of exposure; N + c—estimated number of cases attributable to a certain level of exposure.

**Table 6:** Impact assessment of health outcomes from long- and short-term exposure to O_3_ considering the sum of ozone means over 35 ppb (SOMO35).

Health Endpoint	Timeframe	Age	RR	B + c	N + c
Mortality from RD	Long-term	Total population	1.0290 (1.0103–1.0499)	3.94 (1.43–6.65)	443.23 (160.46–748.56)
	Short-term		1.0060 (1.0000–1.0144)	0.83 (0.00–1.99)	93.37 (0.00–223.97)
Hospital admissions for RD			1.0090 (1.0014–1.0171)	3.59 (0.57–6.73)	403.58 (64.57–756.81)
Mortality from CVD			1.0101 (1.0027–1.0175)	2.03 (0.54–3.50)	228.18 (60.87–393.67)
Hospital admissions for CVD			1.0184 (1.0103–1.0263)	8.84 (5.00–12.54)	994.69 (562.13–1411.28)

RD—respiratory disease; CVD—cardiovascular disease; RR—relative risk; B + c—estimated change of incidence (per 100,000) at a certain category of exposure; N + c—estimated number of cases attributable to a certain level of exposure.

**Table 7:** Impact assessment of health outcomes from short-term exposure to NO_2_ considering the 2021 WHO Air Quality Guideline.

Health Endpoint	Timeframe	Age	RR	B + c	N + c
Hospital admissions for RD	Short-term	Total population	1.0020 (1.0013–1.0027)	0.80 (0.51–1.08)	89.61 (57.25–121.93)

RD—respiratory disease; RR—relative risk; B + c—estimated change of incidence (per 100,000) at a certain category of exposure; N + c—estimated number of cases attributable to a certain level of exposure.

## Data Availability

Data presented in this study are available from the corresponding author upon request.

## Supplementary Materials

The following supporting information can be downloaded at: https://www.mdpi.com/article/10.3390/ijerph20095707/s1, Figure S1: Hourly, monthly, and day-of-the-week variations for PM_2.5_ (a), PM_10_ (b), SO_2_ (c), CO (d), NO_2_ (e), and O_3_ (f) during the sampling period. The shaded areas represent the 95% confidence intervals of the mean; Figure S2: Twenty-four-hour time series for temperature (a), precipitation (b), relative humidity (c), and wind speed (d) during the sampling period; Figure S3: Overall wind rose (a) and monthly wind roses (b) during the sampling period.
