# Supplementary material for: Health Risk Assessment of Exposure to Air Pollutants Exceeding the New WHO Air Quality Guidelines (AQGs) in São Paulo, Brazil
Source: Int J Environ Res Public Health. 2023 May 2;20(9):5707. doi: 10.3390/ijerph20095707 (PMC10177979; doi:10.3390/ijerph20095707)

Health Risk Assessment of Exposure to Air Pollutants Exceeding the New WHO Air Quality Guidelines (AQGs) in São Paulo, Brazil

Caroline Fernanda Hei Wikuats [1,*], Thiago Nogueira [2], Rafaela Squizzato [1], Edmilson Dias de Freitas [1] and Maria de Fatima Andrade [1]

[1] Departamento de Ciências Atmosféricas, Instituto de Astronomia, Geofísica de Ciências Atmosféricas, Universidade de São Paulo, São Paulo 05508-090, Brazil
[2] Departamento de Saúde Ambiental, Faculdade de Saúde Pública, Universidade de São Paulo, São Paulo 01246-904, Brazil
* Correspondence: caroline.wikuats@usp.br

Figure S1 – Hourly, monthly, and day-of-the-week variations for $PM_{2.5}$ (a), PM_{10} (b), SO_2 (c), CO (d), NO_2 (e), and O_3 (f) during the sampling period. The shaded areas represent the 95% confidence intervals of the mean.

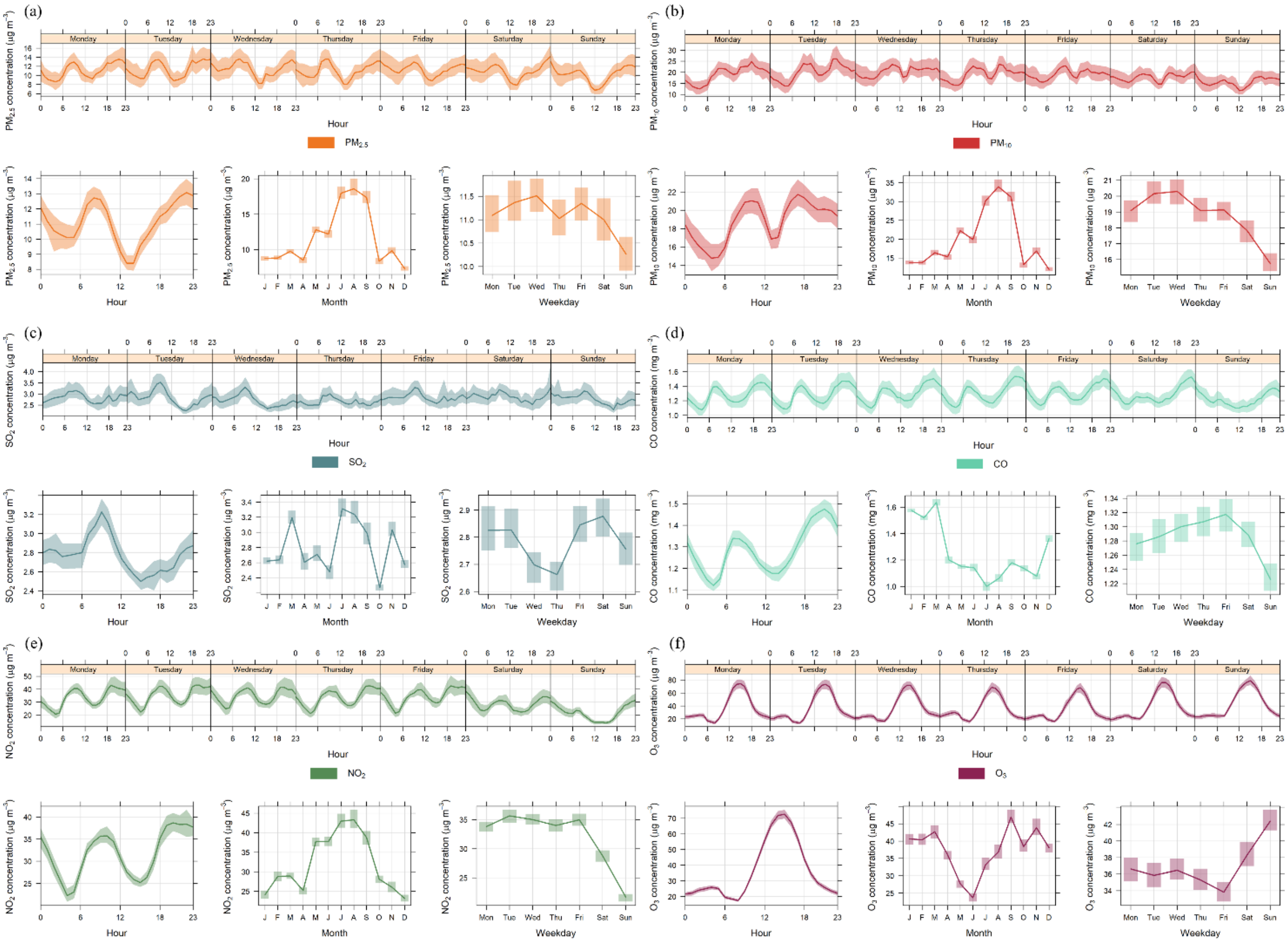

Figure S2 – 24-h time series for temperature (a), precipitation (b), relative humidity (c), and wind speed (d) during the sampling period.

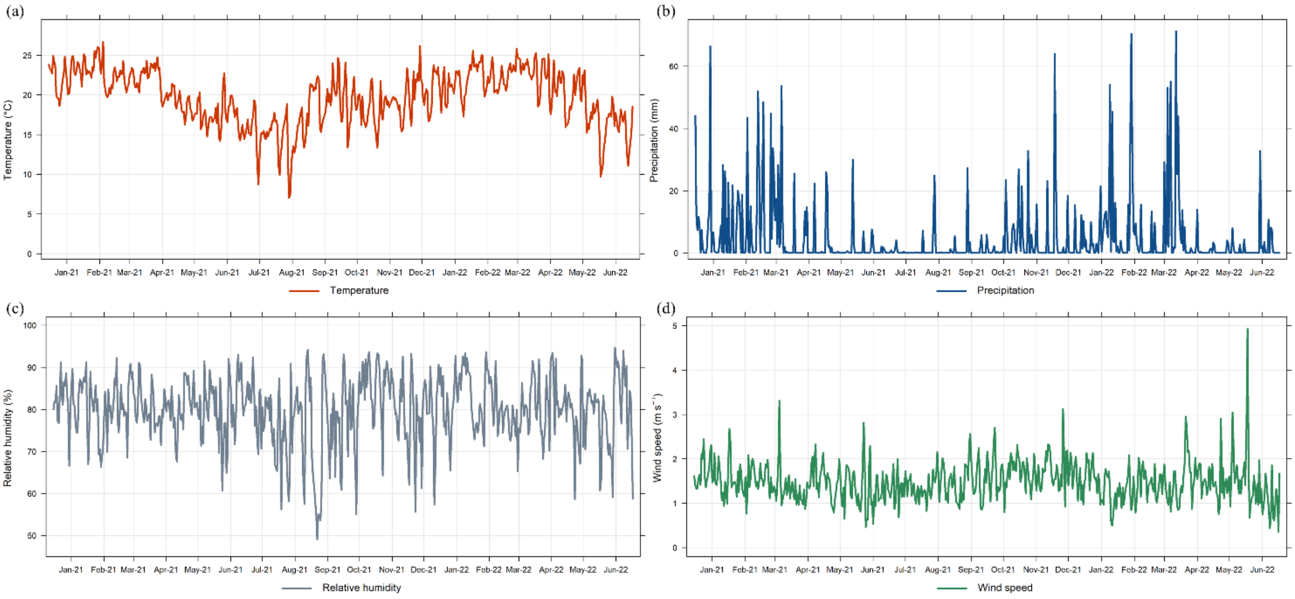

Figure S3 – Overall wind rose (a) and monthly wind roses (b) during the sampling period.

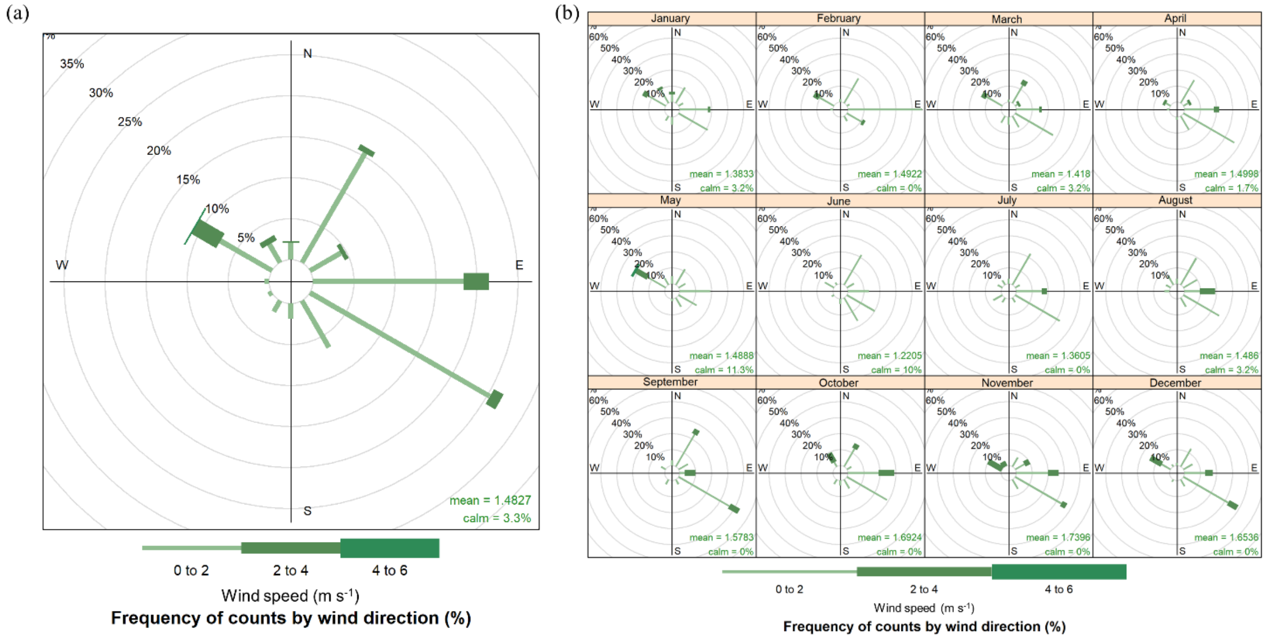

Supplement: Supplementary file 1 [file ijerph-20-05707-s001.zip › ijerph-2281072-supplementary.pdf]
